# Health-related perceptions and drinking motives as actionable targets for precision prevention of high sugar-sweetened beverage intake among Chinese adolescents

**DOI:** 10.3389/fnut.2026.1803900

**Published:** 2026-06-08

**Authors:** Yuting Peng, Junyao Yi, Xiaoyun Yan, Xinyi Chen, Yu Zhou, Zijun Bai, Zuyun Sheng, Dawei Fang, Danyang Wang, Xiaofang Lin, Jieshu Wu

**Affiliations:** 1 Department of Maternal, Child and Adolescent Health, Center for Global Health, School of Public Health, Nanjing Medical University, Nanjing, China; 2 Department of Health Management, Changzhou Health Vocational Technology College, Changzhou, China; 3 Department of Epidemiology, Johns Hopkins Bloomberg School of Public Health, Baltimore, MD, United States; 4 Jiangsu Defuxiyun Health Technology Co., Ltd, Nanjing, China

**Keywords:** adolescent, drinking motives, health-related perceptions, modifiable individual risk factors, nutritional epidemiology, precision prevention, sugar-sweetened beverage

## Abstract

**Objective:**

To quantify the contribution of modifiable individual risk factors (MIRFs)—particularly health-related perceptions and drinking motives—to high sugar-sweetened beverage (SSB) intake among adolescents, and to evaluate their relevance as actionable targets for precision-oriented prevention.

**Methods:**

A school-based survey was conducted among 1,466 Chinese junior high school students to assess SSB consumption and related individual-level factors. Multivariable logistic regression was used to identify factors associated with high SSB intake. To illustrate the practical value of these modifiable factors for identifying and targeting high-risk individuals, a population-based risk stratification approach was applied and evaluated using internal and external datasets. Model performance was assessed using the area under the receiver operating characteristic curve (AUC-ROC), calibration curves, and decision curve analysis (DCA).

**Results:**

Four MIRFs—drinking SSBs as water (OR = 13.00, 95% CI: 7.33–23.07, *R*^2^ = 11.12%), belief in no health effects [“Yes”: OR = 10.61(4.82–23.34); “Unsure”: OR = 2.37 (1.21–4.62); *R*^2^ = 4.99%], a strong desire to consume SSBs (OR = 2.45, 1.49–4.02, *R*^2^ = 1.91%), and drinking due to boredom (OR = 2.27, 1.25–4.13, *R*^2^ = 1.00%)—were strongly associated with high SSB intake (*p* < 0.05), collectively accounting for 71.1% of the model-explained variance. The full model demonstrated good discrimination, with AUCs of 0.858 in the training set, 0.736 in the internal validation set, and 0.751 in the external validation set, along with satisfactory calibration. A simplified model including only these MIRFs showed comparable net benefit in DCA, supporting their potential utility for efficient screening and risk stratification.

**Conclusion:**

Health-related perceptions and non-physiological drinking motives are key modifiable correlates of high SSB intake among adolescents. An MIRF-based screening and targeting approach may support precision prevention strategies and more efficient allocation of intervention resources in school- and community-based public health programs.

## 1 Introduction

Unhealthy diets represent one of the leading preventable risk factors contributing to the global burden of disease (1). Among dietary behaviors, frequent consumption of sugar-sweetened beverage (SSB) is particularly prevalent among children and adolescents. Robust evidence has demonstrated a direct association between SSB intake and elevated risks of obesity, metabolic disorders, and other chronic diseases (2, 3). Given that investing in adolescent health generates a ‘triple dividend’—benefiting health during adolescence, across adulthood, and into the next generation—reducing SSB consumption in this population remains a critical public health priority (4).

Population-level strategies, including SSB taxation (5), health warnings labels, advertising restrictions (6), and sales bans, have been widely implemented to curb SSB consumption (7). While these measures have shown overall effectiveness, their impact is often heterogeneous, and compensatory behaviors—such as shifting purchases to untaxed beverages or regions—have been observed when individual-level determinants are not adequately addressed (5). Notably, evidence suggests that combining structural policies with individual-focused motivational interventions can substantially enhance intervention effectiveness (8), underscoring the continued importance of individual-level factors in shaping SSB consumption behaviors.

Adolescence is a critical developmental period during which health-related perceptions, drinking motives, and habitual behaviors are established and may persist into adulthood (9). Prior research has identified individual characteristics—such as health-related beliefs, consumption habits, emotional needs, and taste preferences—as important drivers of SSB intake (9, 10). However, interventions targeting these factors have shown variable and often limited effectiveness (11–14), partly due to insufficient identification of which modifiable individual factors contribute most substantially to high SSB consumption and therefore warrant prioritization in precision prevention efforts.

Importantly, certain individual-level factors—particularly drinking-related perceptions, non-physiological consumption motives, and situational behaviors—can be assessed with minimal burden and directly targeted through behavioral interventions. These modifiable individual risk factors (MIRFs) may serve as practical and actionable entry points for precision prevention, enabling efficient risk stratification and targeted resource allocation in school-and community-based settings. Therefore, this study aimed to quantify the contribution of MIRFs to high SSB consumption among Chinese adolescents and to translate these factors into actionable targets for precision-oriented prevention strategies.

## 2 Materials and methods

### 2.1 Study design and participants

A school-based cross-sectional study was conducted in 2020 in two cities located in Northern and Southern China, respectively. A cluster sampling approach was applied at the class level within selected schools. Four junior high schools participated, and 2–4 classes per grade were surveyed. The selection of study sites was based on a combination of feasibility and geographic variation considerations. Schools were selected where on-site data collection could be reliably implemented with the support of school staff, and the inclusion of cities from different geographic regions was intended to capture economic and environmental variation in adolescent SSB consumption behaviors.

**Figure 1:**
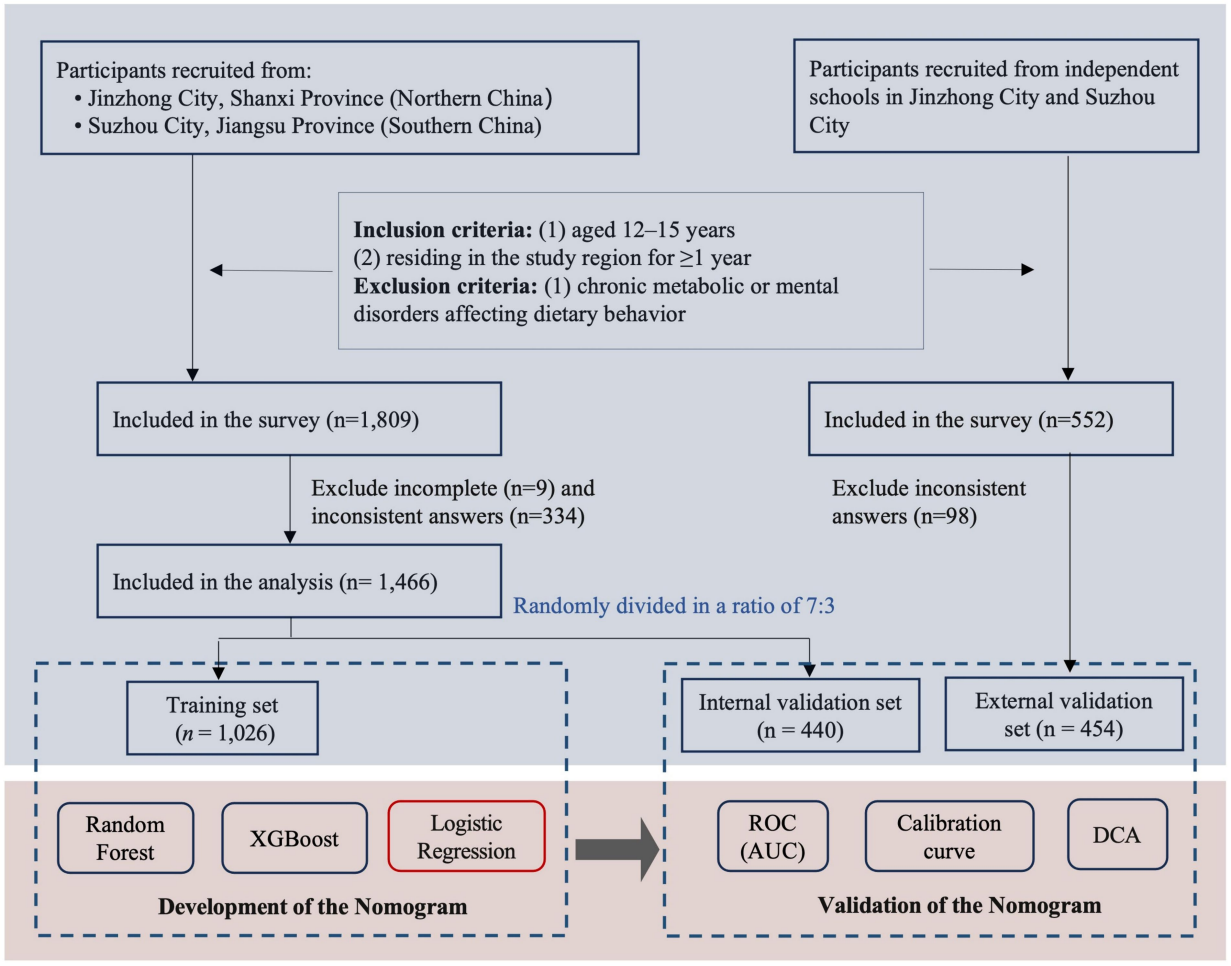
Flowchart of the study process from participant recruitment to nomogram development and validation.

All participating schools were public junior high schools located in suburban areas, and the surveys were conducted in regular classroom settings. Students completed a self-administered questionnaire on demographics, modifiable individual risk factors, and SSB intake, with built-in attention checks to ensure data reliability. To support robust risk stratification, participants were randomly divided into a training set and an internal validation set. An independent cohort was further used for external validation to examine the transportability of findings across settings (Figure 1).

The sample size was not determined *a priori* for prediction modeling, as the primary aim of the study was to examine factors associated with SSB consumption. However, a *post hoc* assessment indicated that the events-per-variable (EPV) ratio was 10.6 in the training dataset, meeting commonly recommended criteria for multivariable logistic regression. The study was approved by the Ethics Committee of Nanjing Medical University (#392, December 21, 2017), and informed consent was obtained from all participants and their parents/guardians.

### 2.2 SSB consumption assessment

SSB consumption was assessed using a validated food group-based food frequency questionnaire (15). Participants were asked, “How often have you consumed SSBs in the past month, excluding 100% fruit juice and artificially sweetened drinks?” along with a detailed list of included and excluded beverage categories and common product examples, according to the definition of SSBs (16). Response options included “daily,” “1–3 times/week,” “4–6 times/week,” “1–3 times/month,” or “<1 time/month” after considering the logical tangent point. To ensure response consistency, a second question—“Do you consume sugar-sweetened beverages three times or more per month?”—was included; conflicting responses were excluded from analysis. High SSB consumption was defined as intake ≥3 times per month. This threshold was determined based on both empirical distribution and prior literature (17), which may differ across populations and contexts.

### 2.3 MIRFs variables and demographics

MIRF variables in this study were conceptualized as modifiable behavioral determinants of SSB consumption, including health-related perceptions, substitution habits, and non-physiological drinking motivations. Their selection was informed by the Theory of Planned Behavior (TPB), particularly its emphasis on attitudes and perceived behavioral control, while also incorporating additional constructs related to habitual behaviors and motivational drivers identified in prior research (10, 18–20).

Six items influencing adolescent beverage intake and feasible for rapid assessment were selected based on literature review and a preliminary survey: (1) Perceived health impact: “Do you think SSBs do not affect health?” (2) Substitution behavior: “Do you drink SSBs as water?” (3) Thirst-driven consumption: “Do you drink SSBs because you are thirsty?” (4) Hunger-driven consumption: “Do you drink SSBs because you are hungry?” (5) Craving-driven consumption: “Do you drink SSBs simply because you want to?” (6) Boredom-driven consumption: “Do you drink SSBs when bored?”

Conceptually, health-related perceptions and substitution-related behaviors broadly reflect attitudinal components, whereas context-related and internally triggered consumption behaviors (e.g., boredom-, craving-or thirst-driven intake) are more closely aligned with perceived behavioral control. Together, these variables represent actionable behavioral entry points that can be targeted in intervention settings.

Responses were categorized as binary or ternary variables according to item design and coded consistently for analysis. All MIRF items demonstrated acceptable test–retest reliability, supporting their suitability for use in population-based behavioral assessment (details shown in Supplementary Table 1).

Gender and family structure (nuclear or non-nuclear) were recorded as categorical variables. Family structure was operationally defined as nuclear (parents living with unmarried children only) or non-nuclear, which included single-parent households, multigenerational households (e.g., three or more generations living together), and other household configurations. Parental educational attainment (illiteracy/primary school, junior high school, or college and above), monthly household income per capita (<¥1,000, ¥1,000–5,000, or >¥5,000), and monthly pocket money (none, ≤¥100, ¥100–200, or >¥200) were treated as ordinal variables. All categorical options were mutually exclusive. Height and weight were self-reported and used to calculate body mass index (BMI, kg/m^2^), which was analyzed as a continuous variable.

### 2.4 Development and validation of the MIRF-based risk stratification tool

The nomogram was developed as a practical screening and targeting tool to stratify adolescents according to their risk of high SSB consumption, with the aim of supporting precision prevention and efficient allocation of intervention resources in school and community settings. Multivariable logistic regression was selected as the primary analytical approach due to its interpretability, and suitability for behavioral risk assessment. Multicollinearity was assessed using the variance inflation factor (VIF), with values <4.0 considered acceptable. To examine the robustness of MIRF importance, random forest and XGBoost models were conducted as sensitivity analyses, focusing on the consistency of feature ranking rather than predictive optimization. Given the comparable or inferior performance of machine-learning approaches and the greater interpretability of logistic regression, the final nomogram was constructed based on the logistic regression model.

The nomogram was generated using the lrm and nomogram functions in R, visually displaying the relative contribution of each determinant and its corresponding point allocation. Individual risk scores were calculated by summing the points across predictors, yielding an estimated probability of high SSB consumption. To quantify variability in risk stratification, the standard deviation (SD) of total points was estimated using 1,000 bootstrap resamples. Discrimination was evaluated using the area under the receiver operating characteristic curve (AUC-ROC), with values >0.70 considered to indicate acceptable screening performance. Calibration was assessed using bootstrap-derived calibration curves and the Hosmer–Lemeshow goodness-of-fit test. Clinical utility was examined using decision curve analysis (DCA), comparing the net benefit of the nomogram with “treat-all” and “treat-none” strategies across a range of threshold probabilities. Utility was assessed in the training dataset and subsequently evaluated in both internal and external validation cohorts to examine transportability of MIRF-based risk stratification across settings.

### 2.5 Statistical analysis

Categorical variables were summarized as percentages, and continuous variables were expressed as mean ± standard deviation. Group differences were assessed using the chi-square test for nominal variables and the Kruskal-Wallis test for continuous or ordinal variables, as appropriate. Univariate and multivariable logistic regression analyses were performed to identify factors associated with high SSB consumption and to explore independent associations across all collected variables. Results were reported as odds ratios (OR) with 95% confidence intervals (CI). Overall model fit was evaluated using Nagelkerke pseudo-*R*^2^.

To develop a parsimonious and interpreted risk stratification tool, a stepwise selection method was applied to identify the final set of predictors. Predictor-specific explained variance (*R*^2^, %) was calculated as the reduction in pseudo-*R*^2^ after removing each predictor from the full model. The contribution of MIRFs was expressed as the proportion of total explained variance attributable to these modifiable factors. Model performance and utility were further assessed in internal and external validation datasets to evaluate the stability and generalizability of MIRF-based risk stratification. All statistical tests were two-sided, with *p* < 0.05 considered statistically significant. Analyses were conducted using R software (version 4.1.0).

## 3 Results

### 3.1 Participant characteristics

After excluding incomplete questionnaires and responses failing predefined consistency checks, 1920 valid questionnaires were retained for analysis. Table 1 presents the characteristics of participants in the training (*n* = 1,026), internal validation (*n* = 440), and external validation (*n* = 454) sets. No significant differences were observed in demographic variables (gender, BMI, household income, family structure, pocket money, parental education), MIRFs, or SSB consumption frequency between the training and internal validation sets (all *p* > 0.05), confirming high comparability. The external validation set had a significantly higher proportion of high SSB consumers (14.8%) than both the training (10.3%) and internal validation (8.6%) sets (both *p* < 0.05; pairwise comparison results not shown). Additionally, students who believed SSBs had no health impact (6.4% vs. 4.0%, *p* < 0.05) or were uncertain (14.3% vs. 9.1%, *p* < 0.05) were more prevalent in the external validation set. No other variables differed significantly between the external validation set and the training or internal validation sets (*p* > 0.05).

**Table 1:** Demographic, MIRF values and SSB consumption characteristics of participants in the training set, internal, and external validation sets.

Variables	Level	Training set	Internal validation set	External validation set
*N*		1,026	440	454
Demographic values				
Monthly pocket money (%)	None	203 (19.8)	93 (21.1)	87 (19.2)
	<¥100	430 (41.9)	191 (43.4)	196 (43.2)
	¥100–200	263 (25.6)	101 (23.0)	112 (24.7)
	>¥200	130 (12.7)	55 (12.5)	59 (13.0)
Gender (%)	Male	539 (52.5)	219 (49.8)	241 (53.1)
	Female	487 (47.5)	221 (50.2)	213 (46.9)
Family structure (%)	nuclear family	766 (74.7)	314 (71.4)	336 (74.0)
	non-nuclear family	260 (25.3)	126 (28.6)	118 (26.0)
Monthly household income per capita (%)	<¥1,000	66 (6.4)	28 (6.4)	23 (5.1)
	¥1,000–5,000	688 (67.1)	294 (66.8)	309 (68.1)
	>¥5,000	272 (26.5)	118 (26.8)	122 (26.9)
Father’s education level (%)	Literacy / primary school	59 (5.8)	18 (4.1)	35 (7.7)
	Junior high school	760 (74.1)	339 (77.0)	331 (72.9)
	College and above	207 (20.2)	83 (18.9)	88 (19.4)
Mother’s education level (%)	Illiteracy/primary school	87 (8.5)	30 (6.8)	41 (9.0)
	Junior high school	717 (69.9)	323 (73.4)	317 (69.8)
	College and above	222 (21.6)	87 (19.8)	96 (21.1)
Height (cm, mean (SD))		163.00 (7.99)	162.73 (8.36)	163.02 (8.30)
Weight (kg, mean (SD))		52.53 (10.82)	52.42 (10.86)	52.99 (11.52)
BMI (kg/m^2^, mean (SD))		19.67 (3.31)	19.72 (3.49)	19.84 (3.51)
MIRF values				
Drinking SSBs as water (%)	No	936 (91.2)	409 (93.0)	412 (90.7)
	Yes	90 (8.8)	31 (7.0)	42 (9.3)
Belief in no health effects (%)	No	892 (86.9)	373 (84.8)	360 (79.3)
	Not sure	93 (9.1)	42 (9.5)	65 (14.3)
	Yes	41 (4.0)	25 (5.7)	29 (6.4)
Thirst-driven consumption (%)	No	644 (62.8)	293 (66.6)	289 (63.7)
	Yes	382 (37.2)	147 (33.4)	165 (36.3)
Hunger-driven consumption (%)	No	998 (97.3)	433 (98.4)	440 (96.9)
	Yes	28 (2.7)	7 (1.6)	14 (3.1)
Drinking due to boredom (%)	No	892 (86.9)	367 (83.4)	384 (84.6)
	Yes	134 (13.1)	73 (16.6)	70 (15.4)
Strong desire to consume SSBs (%)	No	556 (54.2)	230 (52.3)	229 (50.4)
	Yes	470 (45.8)	210 (47.7)	225 (49.6)
SSB consumption frequency (%)	Low	920 (89.7)	402 (91.4)	387 (85.2)
	High	106 (10.3)	38 (8.6)	67 (14.8)

### 3.2 The impact of MIRF on SSB consumption

**Figure 2:**
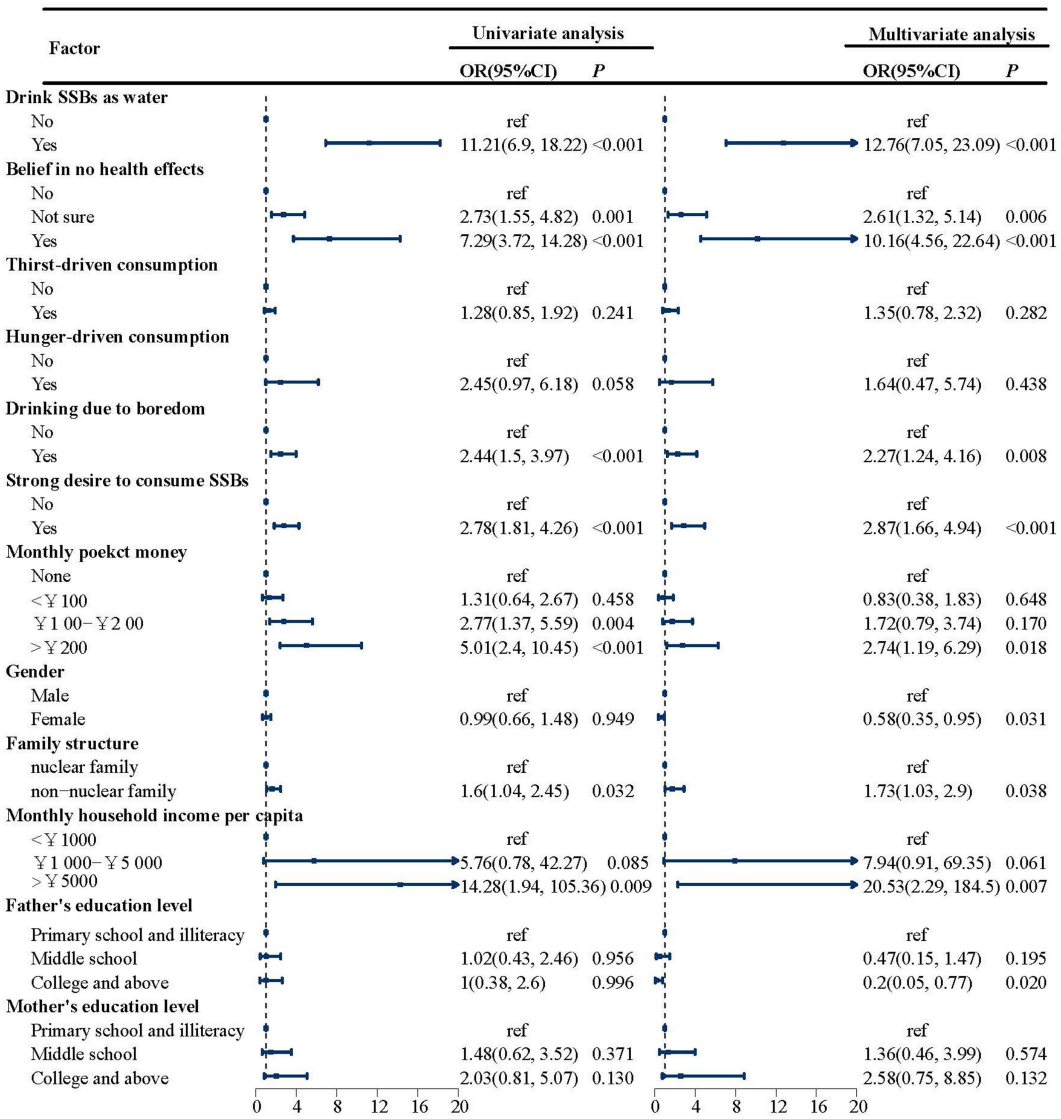
Predictors of high SSB consumption.

Preliminary multivariable analysis including all collected variables identified multiple independent factors associated with high SSB intake (Figure 2). Subsequent stepwise selection was performed to support the development of a risk stratification tool for public health application, identifying eight significant determinants (Table 2).

**Table 2:** Predictors of high SSB consumption identified by stepwise regression analysis.

Variables	OR (95%CI)	*p*	*R* ^2^
Drinking SSBs as water			11.12%
No	ref		
Yes	13.00 (7.33, 23.07)	<0.001	
Belief in no health effects			4.99%
No	ref		
Not sure	2.37 (1.21, 4.62)	0.012	
Yes	10.61 (4.82, 23.34)	<0.001	
Strong desire to consume SSBs			1.91%
No	ref		
Yes	2.45 (1.49, 4.02)	<0.001	
Drinking due to boredom			1.00%
No	ref		
Yes	2.27 (1.25, 4.13)	0.007	
Monthly pocket money			1.86%
None	ref		
<¥100	0.94 (0.43, 2.06)	0.882	
¥100–200	1.89 (0.88, 4.09)	0.103	
>¥200	2.78 (1.22, 6.38)	0.015	
Gender			0.63%
Male	ref		
Female	0.61 (0.38, 0.99)	0.039	
Family structure			0.60%
Nuclear family	ref		
Non-nuclear family	1.7 (1.02, 2.83)	0.040	
Monthly household income per capita			2.89%
<¥1,000	ref		
¥1,000–5,000	6.25 (0.75, 51.86)	0.089	
>¥5,000	15.87 (1.87, 134.72)	0.011	
Model *R*^2^ (Nagelkerke) = 35.2%			

Forest plot displaying odds ratios (ORs) with 95% confidence intervals for predictors identified from univariable and multivariable logistic regression analyses. Variables include sociodemographic characteristics and modifiable individual risk factors (MIRFs).

Among these determinants, four MIRFs together accounted for 71.1% of the explainable variance in high SSB consumption. Partial pseudo-*R*^2^ values were used to estimate the relative contribution of each determinant. Four MIRFs were drinking SSBs as water (OR = 13.00, 95% CI: 7.33–23.07, *p* < 0.001, *R*^2^ = 11.12%), belief in no health effects [“Yes”: OR = 10.61 (4.82–23.34); “Unsure”: OR = 2.37 (1.21–4.62); *R*^2^ = 4.99%], a strong desire to consume SSBs (OR = 2.45, 1.49–4.02, *R*^2^ = 1.91%), and drinking due to boredom (OR = 2.27, 1.25–4.13, *R*^2^ = 1.00%). Demographic determinants included gender, family structure, household income, and monthly pocket money. No collinearity was detected (all VIF < 4; Supplementary Table 2). Collectively, these findings hint at the greater contribution of modifiable individual factors to demographic characteristics and may be useful for identifying adolescents with elevated behavioral risk profiles.

To assess the robustness of MIRF importance, we compared LR with RF and XGBoost as sensitivity analyses. The metrics are summarized in Supplementary Table 3. Sensitivity analyses using RF and XGBoost yielded consistent findings, with MIRFs ranking among the top contributors across models (Supplementary Figure 1). Given its favorable performance and interpretability, LR was selected to construct the final implementable risk stratification tool.

### 3.3 Nomogram construction, discrimination and calibration

**Figure 3:**
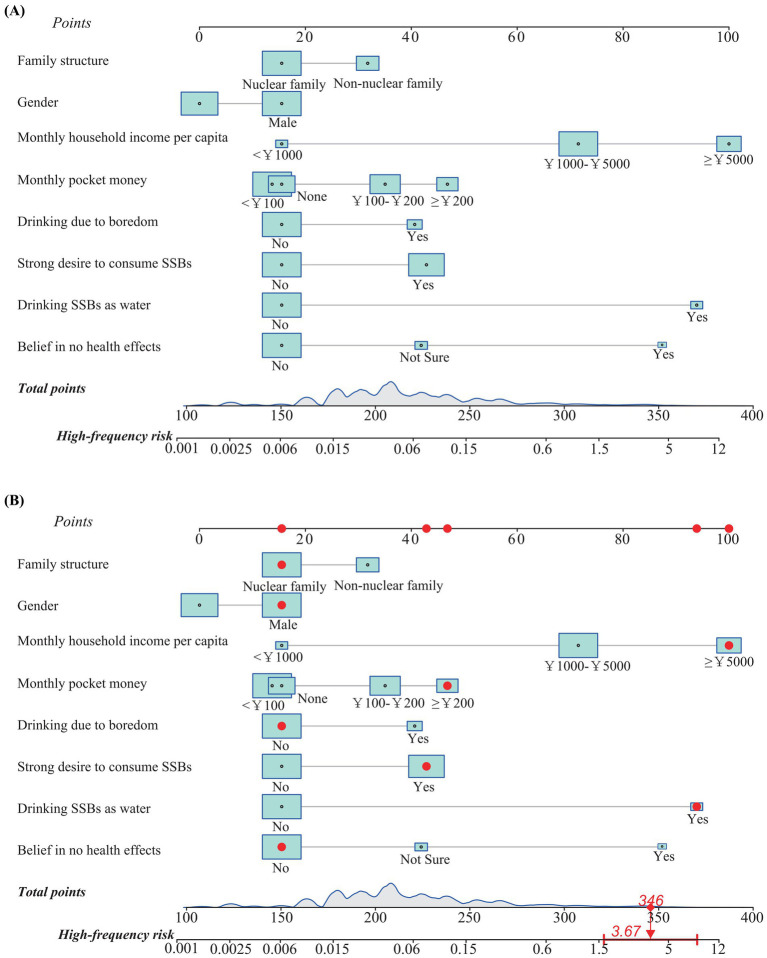
A nomogram for predicting the likelihood of high SSB consumption **(A)** and an example of a nomogram **(B)**.

The final nomogram (Figure 3A) integrates eight determinants, each assigned a specific weight to generate a total points score corresponding to an individual’s risk of high SSB consumption. Based on score dispersion (Supplementary Table 4), the most influential predictors were drinking SSBs as water (SD = 0.131), belief in no health effects (SD = 0.08), income of family per capita (SD = 0.019), and a strong desire to consume SSBs (SD = 0.016). The nomogram provides an interpretable risk score to stratify adolescents by their likelihood of high SSB intake and may help inform prioritized prevention resources in school-and community-based settings (e.g., intensified counseling or behavioral modules). Figure 3B illustrates an example of risk score calculation based on individual MIRF profiles.

**Figure 4:**
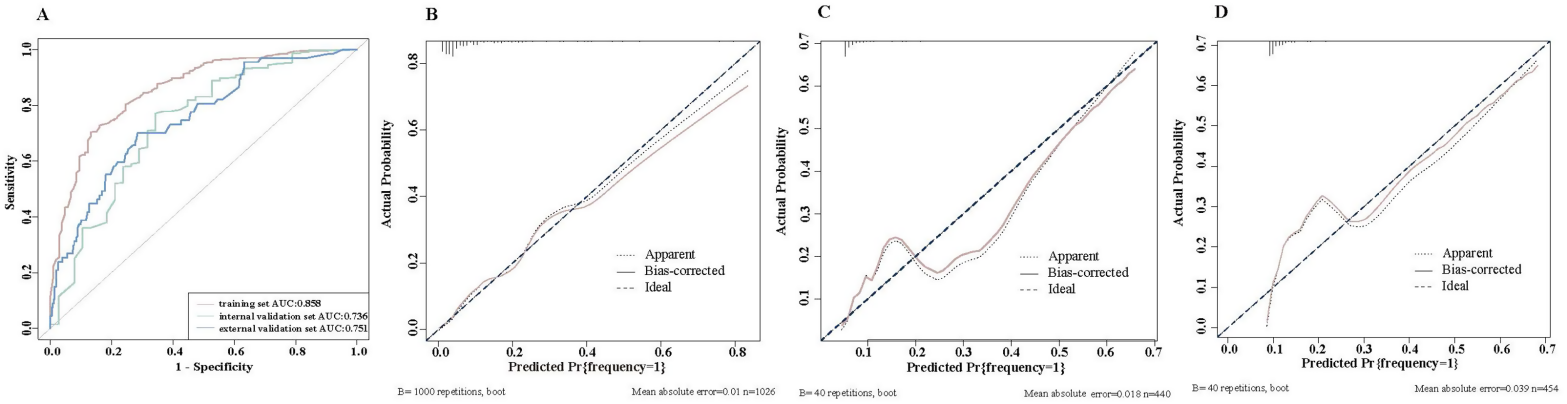
Evaluation of the discrimination, calibration, and clinical utility of the nomogram. **(A)** ROC curves of the nomogram in the training, internal validation, and external validation sets. **(B–D)** Calibration plots of the nomogram in the training **(B)**, internal validation **(C)**, and external validation **(D)**.

**Figure 5:**
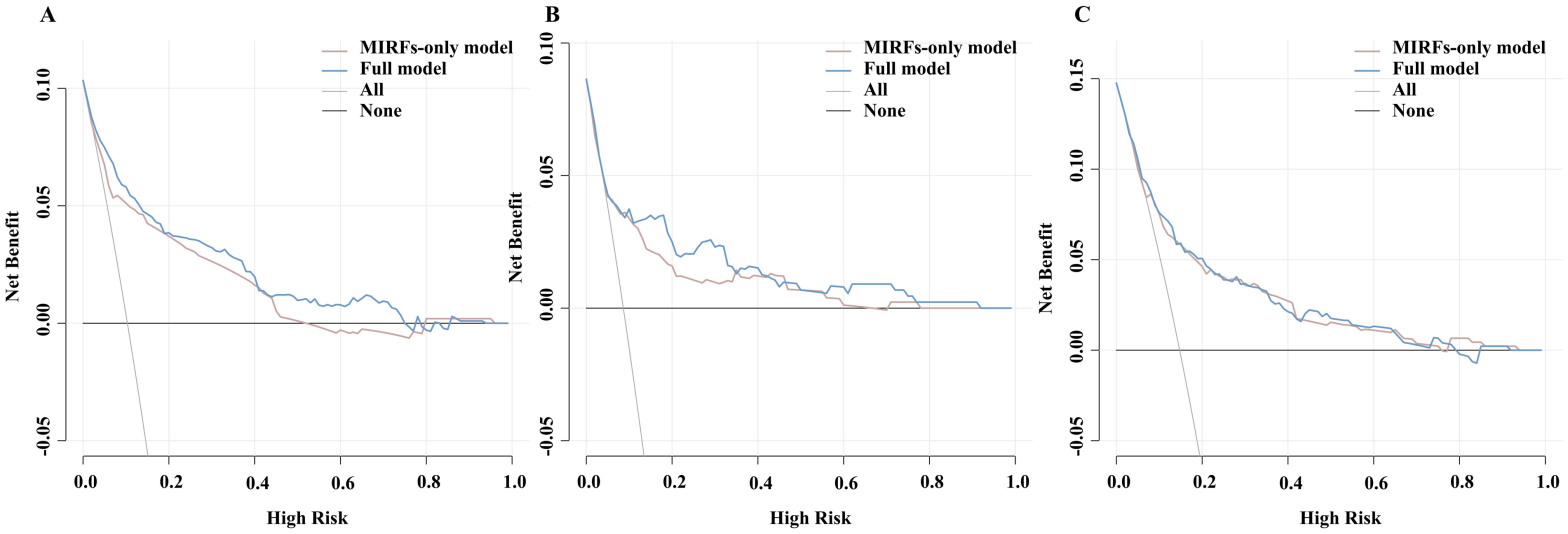
Decision curve analysis of the nomogram for full model and the MIRFs-only model. **(A)** Training set; **(B)** Internal validation set; **(C)** External validation set. Y-axis = standardized net benefit. Dashed grey: treat-all strategy (classify all as high consumers). Solid grey: treat-none strategy (classify none as high consumers). Colored lines: performance of full (red) and MIRFs-only (blue) nomograms across threshold probabilities (0–100%).

The nomogram demonstrated favorable discriminative ability for high SSB consumption, with AUC values of 0.858 (95% CI, 0.821–0.896) in the training set, 0.736 (95% CI, 0.644–0.829) in the internal validation set, and 0.751 (95% CI, 0.688–0.814) in the external validation sets (Figure 4A). Calibration performance was excellent in the training set (calibration slope = 1.0, mean absolute error = 0.011, Hosmer-Lemeshow test *p* = 0.9318). Minor calibration drift was observed in the internal and external validation sets, showing slight deviations from the ideal line but remaining within acceptable limits (Figures 4B–D). As Figure 5 showed, the full nomogram demonstrated superior net benefit compared to both default strategies in the training and validation cohorts.

### 3.4 Performance of the MIRF-only nomogram model

A simplified MIRF-only model incorporating four key variables demonstrated moderate discrimination (AUC ≈ 0.6–0.7) and acceptable calibration (Supplementary Figure 2). Although its discriminative performance was lower than that of the full model, decision curve analysis indicated comparable net benefit across clinically relevant threshold probabilities (Figure 5), supporting its feasibility as a rapid screening tool in resource-constrained settings.

## 4 Discussion

This study quantified the contribution of MIRFs to high SSB consumption among Chinese adolescents and evaluated their utility for precision-oriented risk stratification. MIRFs accounted for 71.1% of the explainable variance in adolescents’ SSB consumption (*R*^2^ = 19.02% out of 26.75% for all predictors). The nomogram demonstrated consistent discrimination across datasets (AUC 0.736–0.858). A simplified model incorporating only MIRFs showed comparable decision-analytic utility to the full model, highlighting their potential as precision-oriented risk stratification and prevention planning tools.

SSB consumption is influenced by both modifiable and nonmodifiable factors. Among nonmodifiable factors, demographic and socioeconomic characteristics have been consistently associated with higher SSB intake. For instance, higher family wealth was a notable independent predictor of SSB intake (OR = 2.15, 95% CI = 1.26, 3.68) (21). Adolescents from single-mother households were more likely to consume SSBs than those from nuclear families (OR = 1.29, 95% CI = 1.06, 1.57) (22). A positive correlation was observed between children’s pocket money and soft drink consumption (*β* = 21.034) (23), and males tended to consume more SSBs than females (24). Our findings align with this body of evidence, confirming that gender, family structure, household income, and pocket money were significantly associated with SSB intake in the studied population. However, because these factors are largely nonmodifiable, they offer limited utility in informing actionable intervention strategies.

In contrast, modifiable factors—particularly those that are easily assessed and directly actionable—have been relatively under-integrated into risk stratification frameworks. Our analysis identified four MIRFs that together explained a substantial proportion of the explainable variance in SSB intake, primarily reflecting drinking-related perceptions, non-physiological consumption motives, and situational behaviors. These factors may represent potentially modifiable entry points for precision-oriented intervention strategies. Specifically, these included drinking SSBs as water, belief in no health effects, a strong desire to consume SSBs, and drinking due to boredom.

“Feeling healthy” is the most influential attitude driving SSB consumption among adolescents across genders (11, 25). Prior research has reported the positive association between adults unaware of specific health risks and higher SSB consumption frequencies (OR = 1.29) (18). Recent studies in adolescents corroborate these findings: those unaware of links between SSB intake and weight gain, heart disease, or cancer had 2.0-, 1.9-, and 2.3-fold higher odds of daily consumption, respectively (26). Parental “healthy” perceptions on SSB also play a critical role in children’s SSB intake (27). Consistent with prior evidence, our findings indicate that the belief that SSBs have no adverse health effects is strongly associated with high SSB consumption among adolescents. This factor may be conceptualized as an absence of core risk perception, reflecting a cognitive blind spot regarding the health consequences of SSB intake. Adolescents holding this “harmlessness” belief exhibited a markedly higher likelihood of high SSB consumption (adjusted OR = 10.61). From a prevention perspective, these findings suggest that addressing such specific misperceptions may offer substantial benefits, supporting a shift from generalized risk awareness toward more targeted corrective education.

Beyond perceptions of health impact, another key attitudinal predictor of soda intake is the belief that SSBs are effective at quenching thirst—a common misconception held by 31.6% of adolescents surveyed (11). A British survey found beverages account for 75% of daily water intake (28). When SSBs are perceived as viable hydration alternatives, adolescent consumption increases significantly (*r* = 0.23, *p* < 0.0001) (11). Consistent with these findings, drinking SSBs as water was strongly associated with high SSB intake (OR = 13.00), accounting for 11.12% of the explained variance. Notably, physiological thirst-driven consumption was not significantly associated (*p* > 0.05). This pattern may reflect a form of hydration-related cognitive substitution, in which SSBs are implicitly regarded as alternatives to water. These findings suggest the importance of moving beyond simple intake restriction toward interventions that improve hydration-related knowledge and behaviors, as exemplified by successful programs such as “Thirsty? Choose Water!” (12, 13, 19).

Based on motivation theory, consumption motives are important factors in shaping adolescents’ dietary behaviors. In this study, a strong desire to consume SSBs and drinking due to boredom emerged as significantly associated with high SSB intake, consistent with evidence highlighting a gap between health risk awareness and actual consumption behavior, suggesting that cognitive knowledge alone is often insufficient to override motivation-driven intake (30). A strong desire to consume SSBs reflects sensory-driven intake rather than physiological need, as conceptualized in established models of food intake regulation (29). Neurobiologically, sugar intake activates dopaminergic reward pathways, which may explain continued consumption despite awareness of health risks (20, 25). Beyond pleasure-seeking, boredom and other negative emotional states represent a compensatory motivational pathway, whereby SSBs function as a short-term coping strategy (31). This “feel better” response has been associated with more frequent SSB intake (*F* = 32.52, *p* < 0.001) (32) and may contribute to neuroadaptations that promote tolerance and habitual consumption patterns (33). Together, these findings suggest that effective interventions should address not only knowledge deficits but also the underlying motivational and emotional drivers of SSB consumption.

Overall, MIRFs were among the most influential factors associated with adolescents’ SSB consumption, explaining 71.1% of the explainable variance and demonstrating utility comparable to the full model. The nomogram translates behavioral risk traits into an individualized risk estimate, enhancing its usability in school-and community-based settings. This tool allows healthcare and school personnel to efficiently identify high-risk adolescents with minimal disruption to routine activities, a key advantage for large-scale school-based screening. Consistent associations between MIRFs and SSB consumption have also been reported across different student populations in previous studies (34). Rather than replacing direct intake assessment, MIRFs provide an efficient framework for identifying high-risk adolescents and their modifiable behavioral characteristics—thereby informing targeted intervention strategies.

## 5 Advantages and limitations

This study presents several notable strengths and practical implications. First, it quantified the relative contribution of MIRFs to adolescents’ SSB consumption and, to our knowledge, developed a novel MIRF-based nomogram for screening and targeting high-risk adolescents in precision prevention strategies. The nomogram demonstrated robust performance, supported by multi-method variable selection and both internal and external validation, indicating good discrimination and generalization. Rigorous reliability testing of key survey indicators further enhanced data quality and model stability. Importantly, this tool enables risk stratification based on individual behavioral profiles, facilitating a shift from broad population-level approaches toward targeted, resource-efficient interventions for adolescents at high risk.

Several limitations should be acknowledged. First, the cross-sectional design precludes causal inference; prospective cohort studies are warranted to assess the temporal and predictive validity of the nomogram. Second, the absence of detailed school-level characteristics (e.g., school environment and resources) may limit generalizability and external validity to other school contexts. Future studies should also incorporate more diverse and systematically sampled school settings to further evaluate and extend the applicability of MIRF-based risk stratification, and context-specific calibration may be required when applying the tool in different populations. Third, as the study was not originally designed for prediction modeling, the sample size was not determined *a priori* based on modeling considerations. Although a *post hoc* EPV assessment suggested adequate model stability, this may reduce the methodological rigor of model development. Finally, the exclusion of respondents with inconsistent responses may limit generalizability to real-world settings. Further work is needed to better understand the causes of inconsistent reporting and to improve data quality in large-scale behavioral surveys.

## 6 Conclusion

Overall, the MIRF-based screening and targeting nomogram provides a practical approach to identifying adolescents at elevated risk of high SSB consumption. By linking modifiable behavioral characteristics to risk stratification, this approach may support more targeted prevention strategies and more efficient allocation of public health resources. Future research should further refine MIRFs and develop corresponding interventions to reduce SSB intake among adolescents.

## Data Availability

The datasets generated and/or analyzed during the current study are available from the corresponding author upon reasonable request and subject to ethical considerations.
