# Supplementary material for: Health-related perceptions and drinking motives as actionable targets for precision prevention of high sugar-sweetened beverage intake among Chinese adolescents
Source: Front Nutr. 2026 Jun 8;13:1803900. doi: 10.3389/fnut.2026.1803900 (PMC13283865; doi:10.3389/fnut.2026.1803900)
Supplement: Supplementary file 1 [file Data_Sheet_1.ZIP › Supplementary/Supplementary Figure 2.pdf]

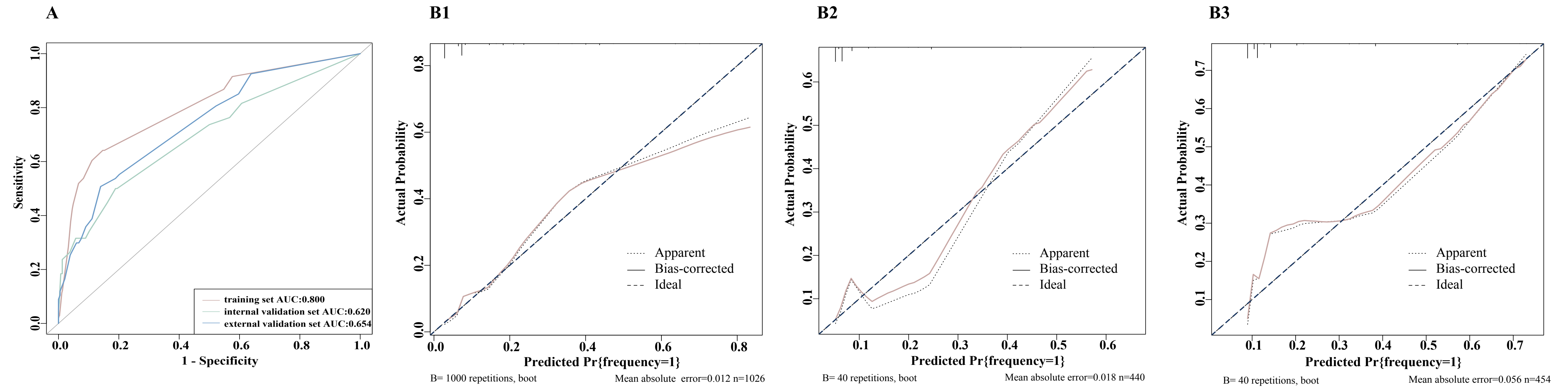

A
B1
B2
B3
Sensitivity
1 - Specificity
training set AUC:0.800
internal validation set AUC:0.620
external validation set AUC:0.654
Actual Probability
Predicted Pr{frequency=1}
Apparent
Bias-corrected
Ideal
B= 1000 repetitions, boot
Mean absolute error=0.012 n=1026
B= 40 repetitions, boot
Mean absolute error=0.018 n=440
B= 40 repetitions, boot
Mean absolute error=0.056 n=454
