# Supplementary material for: Health-related perceptions and drinking motives as actionable targets for precision prevention of high sugar-sweetened beverage intake among Chinese adolescents
Source: Front Nutr. 2026 Jun 8;13:1803900. doi: 10.3389/fnut.2026.1803900 (PMC13283865; doi:10.3389/fnut.2026.1803900)
Supplement: Supplementary file 1 [file Data_Sheet_1.ZIP › Supplementary/Supplementary Table 1.docx]

Supplementary Material

**Supplementary Table 1.** MIRFs Variables and validation measures

| **MIRFs variable** | **Measurement item** | **Response options** | **Scoring** | **ICC** | **Kappa** |
| --- | --- | --- | --- | --- | --- |
| Belief in no health effects | Do you think SSBs do not affect health? | Yes / No / Unsure | 2 / 0 / 1 | 0.612 | 0.856 |
| Drinking SSBs as water | Do you drink SSBs as water? | Yes / No | 1 / 0 | 0.744 | 0.560 |
| Thirst-driven consumption | Do you drink SSBs because you are thirsty? | Yes / No | 1 / 0 | 0.644 | 0.737 |
| Hunger-driven consumption | Do you drink SSBs because you are hungry? | Yes / No | 1 / 0 | 0.518 | 0.643 |
| Strong desire to consume SSBs | Do you drink SSBs simply because you want to? | Yes / No | 1 / 0 | 0.633 | 0.692 |
| Boredom-driven consumption | Do you drink SSBs when bored? | Yes / No | 1 / 0 | 0.653 | 0.713 |
