# Supplementary material for: Health-related perceptions and drinking motives as actionable targets for precision prevention of high sugar-sweetened beverage intake among Chinese adolescents
Source: Front Nutr. 2026 Jun 8;13:1803900. doi: 10.3389/fnut.2026.1803900 (PMC13283865; doi:10.3389/fnut.2026.1803900)
Supplement: Supplementary file 1 [file Data_Sheet_1.ZIP › Supplementary/Supplementary Table 2.docx]

Supplementary Material

**Supplementary Table 2.** Variance inflation factor of all the variance

|  | **Generalized Variance Inflation Factor (GVIF)** | **Degrees of Freedom (Df)** | **Adjusted GVIF [GVIF^(1/(2×Df))]** |
| --- | --- | --- | --- |
| Drinking SSBs as water | 1.15 | 1.00 | 1.07 |
| Belief in no health effects | 1.11 | 2.00 | 1.03 |
| Thirst-driven consumption | 1.27 | 1.00 | 1.13 |
| Hunger-driven consumption | 1.07 | 1.00 | 1.04 |
| Boredom-driven consumption | 1.06 | 1.00 | 1.03 |
| Strong desire to consume SSBs | 1.23 | 1.00 | 1.11 |
| Monthly pocket money | 1.18 | 3.00 | 1.03 |
| Gender | 1.07 | 1.00 | 1.04 |
| Family structure | 1.14 | 1.00 | 1.07 |
| Monthly household income per capita | 1.19 | 2.00 | 1.05 |
| Father's education level | 1.93 | 2.00 | 1.18 |
| Mother's education level | 1.88 | 2.00 | 1.17 |
