# Supplementary material for: Health-related perceptions and drinking motives as actionable targets for precision prevention of high sugar-sweetened beverage intake among Chinese adolescents
Source: Front Nutr. 2026 Jun 8;13:1803900. doi: 10.3389/fnut.2026.1803900 (PMC13283865; doi:10.3389/fnut.2026.1803900)
Supplement: Supplementary file 1 [file Data_Sheet_1.ZIP › Supplementary/Supplementary Table 3.docx]

Supplementary Material

**Supplementary Table 3.**Comparison of predictive performance among Logistic Regression, Random Forest, and XGBoost models

| **Metric** | **Logistic Regression** | **Random Forest** | **XGBoost** |
| --- | --- | --- | --- |
| Sensitivity (Recall) | 0.211 | 0.176 | 0.184 |
| Precision | 0.5 | 0.2 | 0.389 |
| F1-Score | 0.296 | 0.188 | 0.25 |
| Negative Predictive Value | 0.929 | 0.955 | 0.927 |
| RMSE | 0.269 | 0.271 | 0.277 |
| RAE | 0.8919 | 0.6692 | 0.9058 |
