# Supplementary material for: Health-related perceptions and drinking motives as actionable targets for precision prevention of high sugar-sweetened beverage intake among Chinese adolescents
Source: Front Nutr. 2026 Jun 8;13:1803900. doi: 10.3389/fnut.2026.1803900 (PMC13283865; doi:10.3389/fnut.2026.1803900)
Supplement: Supplementary file 1 [file Data_Sheet_1.ZIP › Supplementary/Supplementary Table 4.docx]

Supplementary Material

**Supplementary Table 4.** Standard deviations and score ranges of key predictors in the nomogram

| **Factor** | **SD** |
| --- | --- |
| Drinking SSBs as water | 0.131 |
| Belief in no health effects | 0.080 |
| Strong desire to consume SSBs | 0.016 |
| Boredom-driven consumption | 0.014 |
| Monthly pocket money | 0.014 |
| Monthly household income per capita | 0.019 |
| Gender | 0.004 |
| Family structure | 0.008 |
