# Supplementary figures and images for: Health-related perceptions and drinking motives as actionable targets for precision prevention of high sugar-sweetened beverage intake among Chinese adolescents
Source: Front Nutr. 2026 Jun 8;13:1803900. doi: 10.3389/fnut.2026.1803900 (PMC13283865; doi:10.3389/fnut.2026.1803900)

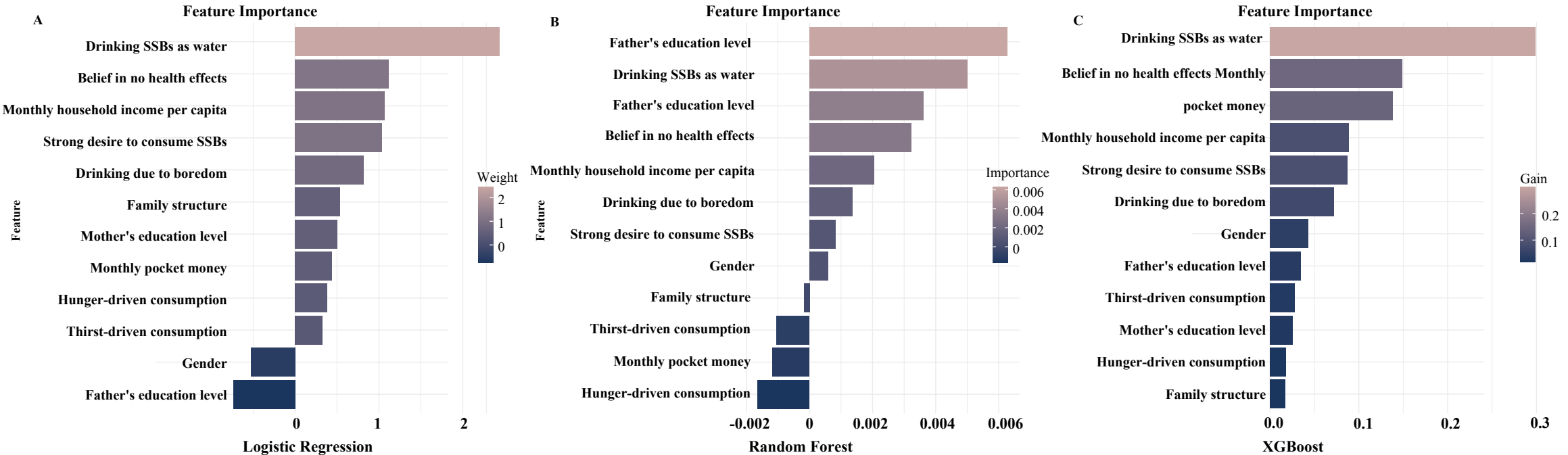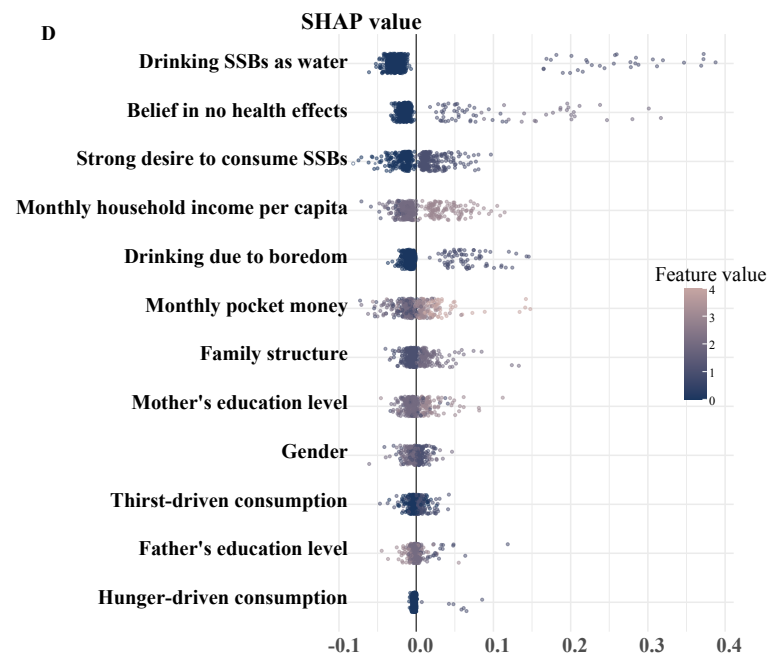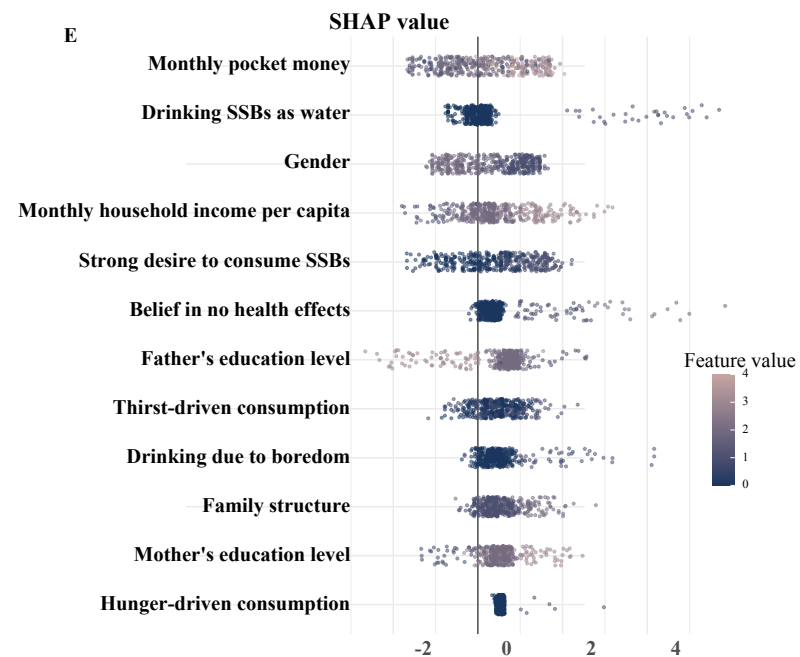

Supplement: Supplementary file 1 [file Data_Sheet_1.ZIP › Supplementary/Supplementary Figure 1.pdf]
